# *TORTOISEV4*: Reimagining the NIH diffusion MRI processing pipeline

**DOI:** 10.1162/IMAG.a.948

**Published:** 2025-12-09

**Authors:** M. Okan Irfanoglu, Amritha Nayak, Paul Taylor, Anh Thai, Carlo Pierpaoli

**Affiliations:** Quantitative Medical Imaging Laboratory, NIBIB, National Institutes of Health, Bethesda, MD, United States; Scientific and Statistical Computing Core, NIMH, National Institutes of Health, Bethesda, MD, United States; Electrical Engineering Department, Catholic University of America, Washington DC, United States

**Keywords:** diffusion MRI, artifacts, distortions, preprocessing, pipeline

## Abstract

Diffusion MRI (dMRI) data suffer from a number of artifacts, including, but not limited to, low SNR, Gibbs ringing, bulk subject motion, within volume motion, eddy-current distortions, susceptibility-induced EPI distortions, and ghost artifacts. Appropriate pre-processing of diffusion-weighted images prior to model fitting is vital for accurate quantitative analysis. Over the years, the nature of dMRI data has evolved (smaller voxel sizes, significantly larger number of volumes and b-values, wider variety of acquisition paradigms, etc.) as have the required processing tools. Additionally, very large multi-site dMRI studies, on potentially uncooperative subjects (young children, geriatric populations, patients with movement disorders, etc.), have increased the necessity for dMRI processing pipelines that are fast, robustly capable of handling a variety of artifacts/distortions, and that have summary reporting capabilities to pinpoint problematic data. *TORTOISE* (Tolerably Obsessive Registration and Tensor Optimization Indolent Software Ensemble) (www.tortoisedti.org) has been redesigned, made adaptable and significantly enriched to meet these needs.

## 1 Introduction

Diffusion MRI (dMRI) has been widely used in clinical and biomedical research applications to infer valuable information about the underlying architectural, microstructural, and compositional features of the human brain (Alexander et al., 2019; Jones & Cercignani, 2010; Pierpaoli et al., 1996). However, obtaining accurate, reproducible, and clinically viable dMRI measures is challenging (Irfanoglu et al., 2021) given that diffusion-weighted images (DWIs), which are collected using Echo Planar Imaging (EPI), are susceptible to various distortions and artifacts (Pierpaoli, 2010; Tax et al., 2022). Therefore, performing a range of preprocessing steps is generally needed to reduce the confounding effects inherent in dMRI acquisitions. Applicable preprocessing methodologies to a dMRI data directly get affected by the employed acquisition scheme where one is informed by the results and performance of the other, necessitating careful planning for an integrated approach.

Pre-processing has been a crucial component in dMRI analysis since its early days, where the deteriorating effects of subject motion and eddy-currents induced distortions were quickly recognized (Bastin, 1999; Haselgrove & Moore, 1996; J. L. Andersson & Skare, 2002; Rohde et al., 2004). Over the years, the pre-processing needs have evolved along with the advancements in acquisition strategies (Sotiropoulos et al., 2013). Today, with a large number of processing steps involved (see Tax et al. (2022) for a comprehensive list), the choice of the pre-processing pipeline, or the steps to apply, may create a sufficiently large variability in the outcome, potentially masking the effects of the biological hypotheses (Botvinik-Nezer et al., 2020). With this realization, several initiatives are underway to determine the effects of preprocessing on dMRI outcome quality with the primary focus being on standardization (Veraart et al., 2023). With the resurgence of very large multi-center dMRI studies, such as the HCP (Sotiropoulos et al., 2013), UK BioBank (Miller et al., 2016), ABCD (Hagler et al., 2019), dHCP (Bastiani et al., 2019), HBCD (Volkow et al., 2021), and many others, the variety in data acquisition protocols and applied pre-processing strategies is only increasing. Even though one of the ultimate goals of dMRI pre-processing is to eliminate or to reduce the effects of scanner, site, or protocol differences, this goal is still very challenging to achieve.

Over the years, the nature of dMRI data has evolved, with smaller voxel sizes, significantly larger number of volumes and b-values, and a wider variety of acquisition paradigms being more and more widely used. The past decade has witnessed a significant number of methodological and technical advancements in post-acquisition strategies regarding nearly every confound involved in dMRI, which aim to further improve the quality of diffusion-weighted images (DWI) beyond what could initially be accomplished.

### 1.1 TORTOISE

*TORTOISE* (Tolerably Obsessive Registration and Tensor Optimization Indolent Software Ensemble) (Pierpaoli et al., 2010) is a diffusion MRI preprocessing package and is rooted in one of the earliest diffusion MRI processing pipelines, as its initial (but publicly unavailable) version utilized code-base from the very first diffusion tensor fitting application (Mattiello et al., 1997; Pierpaoli et al., 1996). These initial versions, which were later enriched with a motion and eddy-currents distortion correction module (Rohde et al., 2004), were primarily used in-house or within the developers’ institution. The first publicly available version of TORTOISE was released in 2010 (Pierpaoli et al., 2010) and evolved over the years, adding the capability to perform reversed phase-encoding, or blip-up blip-down, susceptibility distortion correction (Irfanoglu et al., 2015), achieving good performance compared to other available tools according to independent reviews (Gu & Eklund, 2019).

As the needs for dMRI preprocessing have evolved over the past decade, the entropy of available toolsets has increased as well. *QSIPREP* (Cieslak et al., 2021) and *MRTRIX* (J.-D. Tournier et al., 2019) frameworks aim to reduce the necessity to employ multiple toolsets from different developers by providing a unified platform combining different softwares. We believe that a single code-base providing this comprehensive approach while also being modular will still be beneficial to the community. Therefore, *TORTOISEV4* has been completely redesigned to satisfy what we consider to be the current unmet needs in the field to:

- Incorporate virtually all recent state-of-art advancements in dMRI preprocessing under one umbrella.
- Provide a user-friendly solution package that is easy to call by end-users and a modular design that can be used to embed components into other pipelines by developers.
- Support a variety of dMRI acquisition paradigms, including linear Cartesian q-space sampling to planar and spherical sampling acquisitions.
- Be suitable for deployment for large-scale multi-site dMRI studies.
- Be sufficiently fast to be integrated into a DICOM push-back service.

During the design process, we have collaborated with researchers from the dMRI tractography community, ABCD and HBCD project dMRI acquisition and processing groups to determine other community needs, especially regarding large-scale studies.

### 1.2 *TORTOISEV4* features

One of the design goals of *TORTOISEV4* has been to provide a rich feature set to cater to most processing needs. Table 1 summarizes *TORTOISEV4*’s capabilities along with the most commonly used dMRI preprocessing packages as a reference. In this table, if a cell is blank, this indicates the software does not provide the corresponding functionality, otherwise the cell will have two entries with checkmark and crosses: the first ✓/✗ will indicate whether the solution is proposed by the software’s group (i.e., ✓ : internal, ✗: external) and the second one indicates whether the provided tool is part of the dMRI pipeline (✓) or an independent tool (✗).

**Table 1.** Feature sets of the widely used dMRI pipelines.

	TORTOISE2/3	*TORTOISEV4*	FSL	MRTRIX3	QSIPREP	ExploreDTI	DiPY
ACPC alignment	✗ ✗	✗ ✗	✗ ✗	✗ ✗	✗ ✗	✗ ✗	✗ ✗
Masking	✗ ✓	✗ ✓	✓✓	✓✓	✗ ✓	✗ ✗	✗ ✓
B-matrix check	✗ ✓	✗ ✗		✓ ✗	✗ ✗		✗ ✗
Bias Field Correction				✗ ✓	✗ ✓		✗ ✗
Denoising		✗ ✓		✗ ✓	✗ ✓		✓✓
Noise floor bias	✓ ✗	✓ ✗		✗ ✗	✗ ✗		
Gibbs ringing		✗ ✓		✗ ✓	✗ ✓		✗ ✓
Partial Fourier Gibbs		✗ ✓			✗ ✓		
Grad. Nonlin. estimation		✓ ✗					
Gradwarp		✗ ✓					
Gradnonlin voxelwise Bmat.		✗ ✓					
Inter-volume motion & eddy	✓✓	✓✓	✓✓	✗ ✓	✗ ✗	✗ ✗	✗ ✗
Slice-to-volume motion		✓✓	✓✓	✗ ✓✓*	✗ ✓		✗ ✓
Outlier detection		✓✓	✓✓	✗ ✓✓*	✗ ✓		✗ ✓
Outlier replacement		✓✓	✓✓	✗ ✓✓*	✗ ✓		✗ ✓
Susceptibility Distortions	✓✓	✓✓	✓✓	✗ ✓	✗ ✓	✗ ✓	✗ ✓
Volume-wise Susceptibility			✓✓	✗ ✗	✗ ✗		
Signal Drift		✗ ✓		✗ ✓	✗ ✓	✓✓	✗ ✓
EPI ghosts		**					
Alignment to Anatomical	✗ ✗	✗ ✗	✗ ✗	✗ ✗	✗ ✗	✗ ✗	✗ ✗
Quality Control	✗ ✓	✗ ✓	✓✓	✗ ✓	✓✓		✓✓
Robust model estimation	✓ ✗	✓ ✗		✓✓	✗ ✗		✗ ✗
Tractography			✓ ✗	✓✓	✗ ✗	✗ ✓	✓✓
Diffusion image registr.	✓✓	✓✓	✓✓	✓✓			✗ ✗
Unconventional dMRI acq.		✓✓					

The first ✓ or ✗ indicates whether the implemented solution was proposed by the software’s group. The second one indicates whether the proposed solution is implemented within the pipeline. *: MRTRIX3 provides options to perform slice-to-volume motion correction and outlier detection both with external tools and their proposed methods. **: In development. Diffusion image registr. refers to image registration capabilities while using dMRI information beyond the scalar maps. Unconventional dMRI acq. refers to support for DSI acquisitions and multi-dimensional acquisitions such as planar and spherical encodings.

## 2 Methods

In this section, we will introduce the purposes of each TORTOISE module in the order they are called in the pipeline. In the Supplementary Materials, we provide the implementation details of each module along with a description of user-adjustable parameters.

### 2.1 The DMRI processing pipeline

**Fig. 1.**
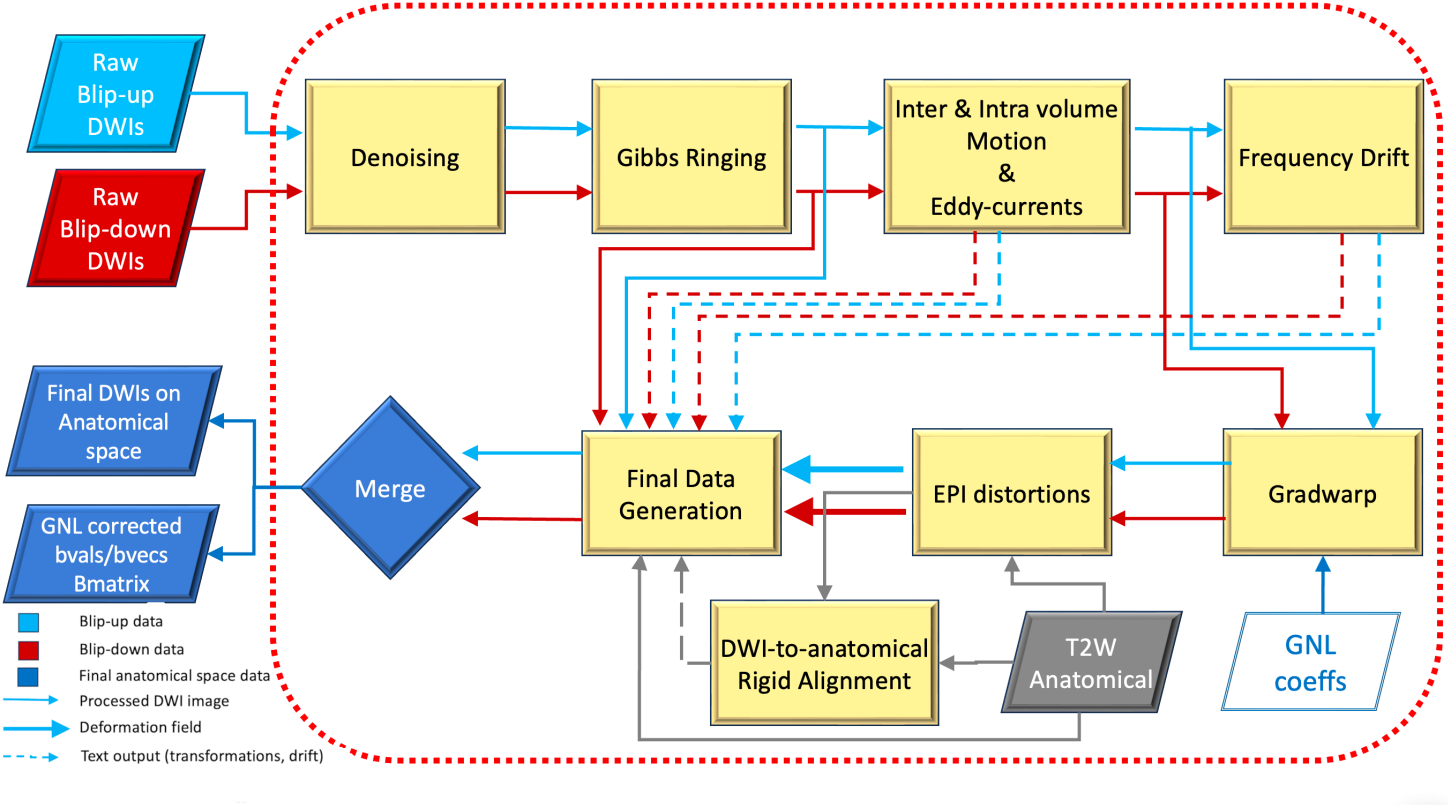
Complete *TORTOISEV4* processing pipeline flow. Processing starts with denoising, followed by Gibbs ringing correction, inter- and intra-volume motion, eddy-currents distortion correction and outlier detection, frequency drift estimation, gradient nonlinearities (GNL) geometric distortion correction, and susceptibility distortion correction. The final stages include DWI-to-anatomical image alignment and the generation of the final data which considers the transformations and signal modulations of all previous steps. For data that do not conform to this pipeline, such as a single phase-encoded data or when gradwarp is applied by the scanner, only the corresponding modules are applied.

The design philosophy behind the new preprocessing pipeline was to combine novel state-of-art techniques that have been proposed in the past decade, and to further develop and improve upon existing methodologies to achieve virtually the best possible data quality. Figure 1 displays the flow of the proposed pipeline, where all individual preprocessing steps are collected and provided under the umbrella of the *TORTOISEV4* diffusion MRI processing package (Pierpaoli et al., 2010). Each preprocessing step, which is detailed below, is a module that can be used directly without involving the entire pipeline, therefore straightforward to be embedded in other dMRI packages. The collection of all these modules constitutes the *TORTOISEV4* pipeline, which are applied in the order depicted below. The strength of using the integrated pipeline instead of cascaded modules is the application of all the transformations with a single interpolation step with minimal degradation of the quality of the output.

The source code, precompiled binaries, and information about compilation or Docker containers can be found at: https://github.com/QMICodeBase/TORTOISEV4.

#### 2.1.1 DWI denoising

Noise in images is an intrinsic problem in dMRI as it relies on measurements of signal attenuation. Heavily diffusion-weighted images are typically noisy and the push toward higher resolution makes this issue even more pressing. Therefore, denoising has become a crucial step in dMRI preprocessing pipelines. However, noise affects images in two aspects: the increase in signal variability and the noise floor bias due to rectification. The term denoising generally refers to the first aspect, with several statistical (Awate & Whitaker, 2007; Baselice et al., 2017), regression-based (Fadnavis et al., 2020), or machine-learning based denoising techniques (Kidoh et al., 2020; Manjon & Coupe, 2019) that had been proposed in the past decade addressing this issue.

Denoising of diffusion-weighted images is the first step in the proposed pipeline, where we opted to use the MP-PCA method (Marchenko-Pastur Principal Components Analysis) (Veraart et al., 2016) as it is currently the most commonly utilized DWI denoising approach in the community.

#### 2.1.2 Gibbs ringing correction

With the local subvoxel shift method (Kellner et al., 2016), Gibbs ringing artifact correction has become an established part of diffusion MRI preprocessing. The initial method, proposed by Kellner et al. (2016) could only be utilized for full k-space acquisitions. Most multi-center studies, however, employ a partial Fourier acquisition, 6​/​8 k-space coverage being the most common, which prevents direct utilization of this method. Recently, Lee et al. (2021) proposed an extension to the local subvoxel shift method, which made it usable with such datasets. Both methods are available in *TORTOISEV4* and the module automatically decides which methodology to use based on the k-space coverage information provided by the data JSON file.

#### 2.1.3 Inter-volume motion & eddy-currents, slice-to-volume, outlier detection & replacement

*DIFFPREP* was the first *TORTOISE* module and its initial version was designed to perform only inter-volume motion and eddy-currents distortion correction (Rohde et al., 2004). This initial motion and eddy-currents correction module had the merit of first underscoring that a simple affine transformation is inadequate to correct for eddy-currents distortions and, indeed, was the first to implement a physics-based quadratic parsimonious model, and it was also the first to apply a proper rotation to bvecs or the Bmatrix to account for motion (Rohde et al., 2004). With the release of the Young Adult Human Connectome Project (HCP), *FSL*’s (Smith et al., 2004), other needs have emerged such as within volume motion correction (i.e., slice-to-volume motion), outlier detection, and replacement. FSL’s excellent Eddy module (J. L. Andersson & Sotiropoulos, 2016) with its extensions (J. L. Andersson et al., 2017, 2018; J. L. R. Andersson et al., 2016) has proposed strategies to address these additional needs; however, Eddy requires data to be structured in a particular way, that is, the data need to be sampled in discrete shells, with a large number of images for each shell.

The *DIFFPREP* module of *TORTOISE* has been completely redesigned, enriched, and optimized with new feature-sets to accommodate these additional dMRI processing needs. It was designed to be applicable to all dMRI datasets, without requiring a discrete shell experimental design and the ability of working with essentially any arbitrary combination of diffusion-weighted images, including Cartesian sampling paradigms such as Diffusion Spectrum Imaging (Wedeen et al., 2000). With shelled data, it does not suffer from uneven distributions of volumes per shell, such as the typical low number of volumes for b ∈  [50−700] $s/mm^{2}$.

**Fig. 2.**
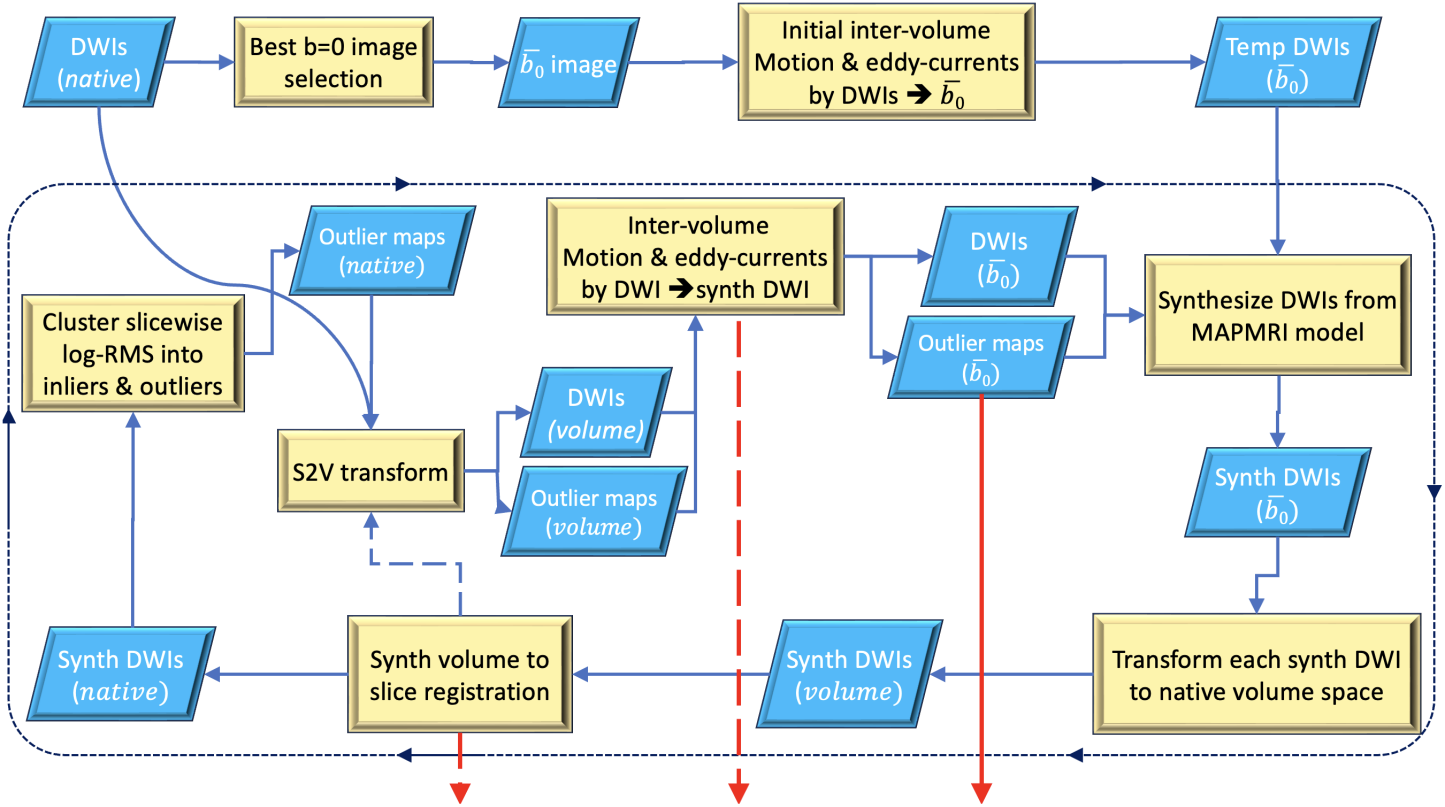
*TORTOISEV4*’s inter-volume motion, eddy-currents distortion, within-volume motion, and outlier detection pipeline. Straight lines represent the generated temporary images, and the dashed lines are text transformations. The red arrows represent the outputs of the pipeline, which are slice-to-volume transformation text files, inter-volume motion and eddy-currents parameters, and the slicewise outlier probability maps. *synth* refers to synthesized predicted data.

The flowchart for the complete module is depicted in Figure 2. Depending on the specified parameters, only a few or all the sub-modules might be applied; for instance, *DIFFPREP* can be asked to perform an operation as simple as an inter-volume motion correction with B-matrix reorientation or eddy-currents distortion correction (for phantoms) or the complete pipeline with slice-to-volume alignments and outlier detection. The detailed description of the complete pipeline is as follows:

The module starts by extracting the “best b = 0 ” (i.e., the least artifactual) image out of the dataset. This is performed in a similar manner to the processing pipeline developed for the “Developing Human Brain Connectome” (dHCP) project (Bastiani et al., 2019), as all the $b=\;\;0s/mm^{2}$ images are first rigidly registered to the first one in the dataset. A patch-based cross-correlation metric (window size: 5 × 5 × 5 ) is computed between all pairs of images, and the pair with the highest similarity is averaged to generate the best image, $\bar{b_{0}}$.

For the description of the *DIFFPREP* pipeline below, all the generated images will be denoted as “datatype_dataspace”, with “datatype ∈  {raw, snyth}” and “dataspace $\in \;\;\left\{native,\;volume,\;\bar{b_{0}}\right\}$, native representing the very native space of the acquisition including the slice-to-volume misaligments, volume representing the space of a volume after slice-to-volume correction is performed, and $\bar{b_{0}}$ is the final corrected space.

0. *Initial inter-volume motion & eddy*: All the raw DWIs (raw_native ) are registered to the $\bar{b_{0}}$ image, including the high b-value volumes, with a quadratic/cubic transformation, specifically designed to represent the physics of eddy-currents distortions (Barnett et al., 2014; Rohde et al., 2004). These transformations also include the inter-volume motion components ($\Rightarrow raw\text{\_}\bar{b_{0}}$)

1. *Signal prediction*: After this initial registration, a MAPMRI propagator-based signal model (Özarslan et al., 2013) of degree 4 is fitted to this temporary data, and the entire DWI set is regenerated from the model ($\Rightarrow synth\text{\_}\bar{b_{0}}$).

2. *Synthetic image transformation to native-space*: Each synthetic volume is then brought back to its volume-native space, where within-volume motion estimation is performed (⇒synth_volume ):

3. *Volume-to-Slice Registration*: For each volume v, and each multi-band slice group, the synthetic volume $synth\text{\_}volume^{v}$ is quadratically registered to the corresponding slice-group of $raw\text{\_}volume^{v}$ to generate $synth\text{\_}native^{v}$, that is, a synthetic dataset at the very native space of the acquisition (⇒synth_native ).

4. *Outlier detection*: For each volume v within a shell, each multi-band slice group s, the root-mean-squared (RMS) difference between the real and synthesized images are computed at the very native space as: $RMS_{s}^{v}={\left(\sum_{ijk}{\left(synth\text{\_}native_{s}^{v}-raw\text{\_}native_{s}^{v}\right)}^{2}\right)}^{1/2}$ . Inspired by the outlier detection method described by (Christiaens et al., 2021), the log-RMS values are clustered into four clusters (instead of two representing inliers and outliers) with the Expectation-Maximization method. These clusters are subsequently merged, beginning with the two lowest RMS mean clusters, until only two clusters are left with the lower RMS cluster, that is, inliers, accounting for at least 65% of the data (a heuristic threshold assumption for the proportion of “good” data points). This approach of outlier detection was empirically determined to be more robust than simple Z-scores or the two-cluster approach, especially when the outliers exhibit a bimodal or non-Gaussian distributions, when different types of outliers are present on the slice. Using these distributions, the probabilities of each slice being an inlier are computed and forwarded to subsequent stages (⇒weight_native ).

5. *Transformation to* $\bar{b_{0}}$ *space*: The raw DWIs raw_native , and the weight images weight_native are transformed to the $\bar{b_{0}}$ space using the volume-to-slice and inter-volume motion & eddy transformations ($\Rightarrow raw\text{\_}\bar{b_{0}}$, ⇒ $weight\text{\_}\bar{b_{0}}$).

6. *Inter-volume motion & eddy*: Each $raw\text{\_}native^{v}$ is again quadratically (or cubicly) registered to its corresponding $synth\text{\_}{\bar{b_{0}}}^{v}$, while also using the outlier information as a mask, to generate a new iteration of ($\Rightarrow raw\text{\_}{\bar{b_{0}}}^{v}$).

7. This new temporary set is forwarded to Step 1, where the MAPMRI module also utilizes the inlier weights $weight\text{\_}\bar{b_{0}}$ during the estimation process. Steps 1-6 are iterated until convergence. For most datasets, four iterations were determined to be sufficient.

The MAPMRI model used in this framework models the average diffusion propagator. Therefore, the signal resynthesis using the MAPMRI model can be considered as an interpolation technique in the q-space.

#### 2.1.4 Signal drift correction

For long acquisitions where a single series dictates the collection of a large number of volumes, a drift in center frequency might occur during the acquisition, which can cause a shift in images and a change in the maximum gain. The shift is typically corrected during motion correction. For the change in gain, we opted to implement the linear and quadratic signal estimation method proposed by Vos et al. (2017).

#### 2.1.5 Gradient nonlinearities - CreateGradwarpField, FitNIFTIIso

The *DRBUDDI* method (Irfanoglu et al., 2015) used for susceptibility distortion correction can employ an undistorted anatomical T2W image to further improve distortion correction quality. For most acquisitions, the default approach to gradient nonlinearity induced distortion correction, generally referred as Gradwarp (Glover, 1986), is to use this correction for anatomical images but not for DWIs. This creates a geometric inconsistency between the b = 0 images to be used for susceptibility distortion correction and the anatomical images. Therefore, we provide a module that can generate an ITK compatible gradwarp displacement field, from manufacturer-provided gradient nonlinearity coefficients. This field is applied to the b = 0 images after signal drift correction and prior to susceptibility-induced distortion correction in the pipeline.

Gradient nonlinearity spherical harmonics coefficients are generally considered proprietary information by manufacturers and not all users have direct access to this information. However, if a suitable phantom is available (Pierpaoli et al., 2009), these coefficients can be estimated along with any miscalibrations in gradient amplifiers (Barnett et al., 2021; Rogers et al., 2018; Tan et al., 2013). In addition to the application of gradient nonlinearities, *TORTOISEV4* also provides tools to estimate these coefficients from an isotropic diffusion phantom.

#### 2.1.6 Susceptibility distortion correction

Susceptibility distortion correction algorithms have seen tremendous improvements since the early approaches based on field-mapping (Jezzard & Balaban, 1995) or elastic registration (Kybic et al., 2000; Wu et al., 2008). The reversed phase-encoding (or blip-up blip-down) based techniques have been shown to perform significantly better than these classical approaches, and TOPUP (J. L. Andersson et al., 2003) from the *FSL* package has been one of the pioneers of this correction strategy, which necessitates a planned integrated design between acquisition and processing.

The past decade has witnessed the emergence of many blip-up blip-down correction algorithms to further improve data quality. The reader is encouraged to refer to (Tax et al., 2022) for a list and details of these methodologies. Among these, *DRBUDDI* (Irfanoglu et al., 2015) has been recently determined to perform generally superior to the alternatives in the human brain by an independent set of researchers (Gu & Eklund, 2019). It also has been applied to other organs (Wade et al., 2024). *DRBUDDI*’s improved performance can be attributed to the following feature set:

- *DRBUDDI*’s deformation model is diffeomorphic and fully flexible, with up and down transformations having a slight freedom to deviate from the anti-symmetry principles imposed by blip-up blip-down schemes, which may be necessary for datasets with significant motion or different shim settings between the up and down data.
- In addition to using the blip-up and blip-down b = 0 images for correction (as performed by other tools), *DRBUDDI* can utilize anatomical images with T2W contrast to further improve, constrain, and regularize the distortion correction process.
- The b = 0 and anatomical images have homogeneous contrast within the white matter. Therefore, in small regions that consist of several white matter pathways, such as the Pons, distortion correction strategies that solely rely on b = 0 contrast lack the information to compute an accurate deformation. For data with DWIs in both the blip-up and -down directions, such as HCP, *DRBUDDI* can also automatically utilize the anisotropy and directional information, as provided by the diffusion tensor, to further improve the correction quality.

*DRBUDDI* employs the SyN transformation model (Avants et al., 2008) in a multi-stage setting, with each stage varying in their down-sampling and image smoothing factors, deformation field smoothing kernel sizes, and employed image similarity metrics for a total of 28 stages. The image similarity metrics employed by *DRBUDDI* are as follows:

- *Mean-Squares-Jacobian (MSJac)*: Similar to the traditional blip-up blip-down approaches, this metric computes the similarity between transformed blip-up, blip-down b = 0 images with Jacobian signal manipulation. The gradients to be used by the optimizer are computed analytically from a window of 3 × 3 × 3 , where the center voxel guides the displacements for shape matching, and the voxels along the phase-encoding direction within the kernel affect the Jacobian gradients.
- *Cross-Correlation (CC)*: This metric computes the similarity of blip-up and blip-down fractional anisotropy maps in case both sets have sufficient diffusion information to estimate a diffusion tensor.
- *Mean-Squares-Tensor*: Similarly, this metric computes the directional similarity of blip-up blip-down diffusion tensors (Irfanoglu et al., 2016; Zhang et al., 2006).
- *Cross-Correlation-Jacobian (CCJac)*: This metric computes the similarity of transformed and Jacobian signal manipulated blip-up and blip-down b = 0 images with a distortion-free anatomical image at abstract timepoint t = 0.5 . In this abstract domain, t = 0 represents the space of the blip-up image, t = 1 the blip-down image, and t = 0.5 the corrected, or distortion-free space.
- *Cross-Correlation-Structural-K (CCSK)*: The estimation of transformation Jacobians can be inaccurate and noisy. Following the formulation of Bowtell et al. (1994), the original *DRBUDDI* formulation (Irfanoglu et al., 2015) proposed this metric, where the signal redistribution of the transformed images can be performed without involving the computation of Jacobians. This metric computes the similarity of the combined up and down b = 0 images that we denote K, with the distortion-free anatomical image at t = 0.5 .

Varying combinations of each of these image similarity metrics are employed at different stages of *DRBUDDI*, with MSJac metric being favored at earlier stages and tensor and anatomical image-based metrics being favored at latter ones. The reader is referred to the Supplementary Materials Section SA1 and SA2 for details.

#### 2.1.7 Rigid alignment to an anatomical T1W or T2W image

Rigid alignment of a distortion-corrected b = 0 image to an anatomical image is generally considered a straightforward operation. However, we have observed a large number of failed registrations with subjects from different multi-center studies, especially while using T1W anatomical images. To achieve a fully-automated and robust rigid registration, once again, we utilized the ITKV5 and ANTS (Avants et al., 2011) framework in a multi-stage setting. The registration is performed four times: twice with local Cross-Correlation and twice with Mutual Information metrics with different parameter sets using a Conjugate Gradient Optimization scheme. In case the standard deviation of the four computed transformations is larger than a threshold (THR = 1mm at the periphery of the image), a multi-start rigid registration framework is carried out with starting angles iterating by $30^{o}$ over all axes. The resulting transform of the multi-start registration is validated by performing an inverse registration, that is, anatomical to b = 0 , and checking the inverse consistency of the two transforms.

#### 2.1.8 Final DWI generation

The last module in TORTOISE pipeline is to generate the final processed data by taking into account all the previously computed transformations. In this step, for a given DWI volume j, all the transformations corresponding to $Vol^{j}$ are first combined into a single deformation field. Then, the denoised and Gibbs ringing corrected version (if requested) of $Vol^{j}$ is transformed onto the final anatomical template space with the interpolation technique described in the Supplementary Materials (SA4). At this point, this transformed data ($Vol^{j}(\phi )$ ) is not Jacobian manipulated; therefore, blip-up and blip-down data are not compatible due to differences in signal pile-ups and expansions. A temporary signal-corrected version of these transformed images is then created by multiplying them with the overall transformation Jacobians ($Vol^{j}(\phi )\left|\phi \;\right|$). Then, both blip-up and blip-down data are used along with binary outlier masks (not floating point weight images) to re-estimate the predicted signals.

In case the B-matrices of up and down datasets are near identical, the final non-Jacobian transformed images ($Vol^{j}(\phi )$ ) are then combined into a single dataset using Equation 4 in (Irfanoglu et al., 2015). In case the two B-matrices are different, the user has the option to output the Jacobian manipulated images as two separate datasets or a single concatenated set. Either way, the outlier signal in this dataset is replaced with the previously predicted signals to generate the final dataset.

#### 2.1.9 Gradient nonlinearities - diffusion sensitization

Gradient nonlinearities, which result in spatially varying diffusion sensitization (i.e., voxelwise bvals/bvecs), can be significant in strong gradient systems and can cause lack of harmonization with data from multi-center studies. The original Human Connectome Project dataset provided this information in the form of a “gradient_deviation” image, where each voxel contained a 3 × 3 affine matrix multiplier to the prescribed bvecs to generate the effective bvals/bvecs.

*TORTOISEV4* pipeline was designed to output this information in three ways:

1. Gradient_deviation tensors: Similar to the original HCP, we provide gradient deviation tensor images for researchers to compute the effective voxelwise diffusion sensitization. These images, however, are computed analytically from the manufacturer-provided spherical harmonics coefficients (Barnett et al., 2021); therefore, they do not suffer from numerical differentiation and interpolation issues.

2. Voxelwise-Bmatrices: Gradient_deviation tensors assume no significant motion among the volumes within the dataset. In order to also consider the interaction of gradient nonlinearities with motion, our pipeline has the capability to output voxelwise B-matrices (or voxelwise bvecs/bvals). However, this option, even though the most accurate, increases the data size and storage requirements significantly (6 ×  DWI data size).

3. Gradient Deviations First-Order Approximation (GDFA) (Irfanoglu et al., 2024): Many software packages are not designed to handle gradient deviation tensors or voxelwise B-matrices in their fitting process. Additionally, for deconvolution-based models (Descoteaux et al., 2009; J. D. Tournier et al., 2004), their use is impractical due to the need to recompute response functions voxelwise, which is computationally very expensive. Therefore, it is desirable to provide a dataset with a constant Bmatrix, which also includes gradient nonlinearity effects. GDFA data serves this purpose.

The GDFA data is generated as follows (Irfanoglu et al., 2024): First, all diffusion signals were voxelwise recomputed for nominal b-value by computing the correct Apparent Diffusion coefficient (ADC), based on the effective and nominal b-values assuming mono-exponential decay. Second, a voxelwise spherical harmonic (SH) decomposition (l = 10 ) is performed on each shell using the effective gradient directions. The final corrected signal is regenerated using these SH coefficients and the prescribed, spatially invariant gradient orientations. This regenerated dataset can directly be used by any diffusion model or software as no additional nonlinearity information needs to be considered.

In case gradient deviation tensors or voxelwise Bmatrices are output by the pipeline, all the diffusion model estimation modules (tensor and MAPMRI) are designed to make use of this information.

#### 2.1.10 Quality control

All the intermediate images are saved in a temporary folder for quality control purposes, which the user has the option to keep or delete after processing. Quality control reports, sample images with underlays/overlays are saved in HTML format through *AFNI*’s (Cox, 1996) reporting mechanisms, which the users can examine in a browser for a fast and efficient data curation process.

### 2.2 Validation

One of the primary goals of this manuscript is to showcase the feature set of *TORTOISEV4*. Naturally, some of these features require systematic validation. Most of the modules implemented in *TORTOISEV4* have been systematically and extensively validated in their corresponding papers, including inter-volume motion and eddy-currents correction (Rohde et al., 2004), susceptibility distortion correction (Irfanoglu et al., 2015), dMRI registration (Irfanoglu et al., 2016), gradient nonlinearity (Barnett et al., 2021) modules from the authors of this work and denoising (Veraart et al., 2016), gibbs ringing (Kellner et al., 2016; Lee et al., 2021), and frequency drift (Vos et al., 2017) correction modules by external developers.

However, the module that requires a full systematic validation is the intra-volume motion and outlier detection/replacement algorithm, which is novel. As the goal of this manuscript is to describe *TORTOISEV4* as an ensemble, a full-scale systematic pipeline comparison is beyond the scope of this work, as it would require contributions from individual pipeline developers and should be more a community effort with external raters assessing processing quality. We are currently in the process of performing this validation for a separate research paper. However, in this paper, we still wanted to provide a sample set of results from this module and a limited set of validation (in the Supplementary Materials) for completeness. These results aim to give potential users an idea of TORTOISE’s capabilities and should not be interpreted to assess relative pipeline performances.

For the validation of the overall pipeline, all results were first visually examined. For quantitative validation, due to the lack of ground-truth images, our strategy was to evaluate within-subject scan variability. When longitudinal scans of the same adult subject are performed within a short succession, biological variabilities can be assumed to be negligible. Therefore, any differences between the scans can be attributed to experimental variability and processing differences. A processing pipeline that produces “more similar” images from longitudinal scans can be hypothesized to perform better. In this work, we analyzed the longitudinal variability of FA and MD modalities.

### 2.3 Datasets

To test the proposed *TORTOISEV4* pipeline, several datasets with different properties were utilized:

#### 2.3.1 Motion correction performance data

In order to assess *TORTOISEV4*’s performance with heavily artifactual data, we employed nine subjects from the Developing Human Connectome Project (Bastiani et al., 2019) dataset (results from only a single subject are showcased) and a single subject from the Adolescent Brain Cognitive Development project (Hagler et al., 2019). To recap, the dHCP dataset was acquired on a 3T Philips Achieva scanner equipped with a dedicated 32-channel neonatal head coil with a multiband factor of 4 (TR = 3800 ms, TE = 90 ms). A total of 64 interleaved overlapping slices (in-plane resolution = 1.5 mm, thickness = 3 mm, overlap = 1.5 mm) were acquired for each single volume to obtain an isotropic voxel resolution of 1.5 mm after super-resolution. A total of 300 volumes (20 $=\;0s/mm^{2}$) was collected for each subject, distributed over four phase-encoding directions: Anterior-Posterior (AP), Posterior-Anterior (PA), Right-Left (RL), Left-Right (LR) using three different b-value shells (400, 1000, and 2600 $s/mm^{2}$). The ABCD subject was acquired on a Siemens 3T Prisma scanner, multiband factor 3, 1.7 mm isotropic resolution and b-values of (500, 1000, 2000, and 3000 $s/mm^{2}$).

*TORTOISEV4* outputs were visually compared to the raw unprocessed data to illustrate data quality improvements.

#### 2.3.2 Susceptibility distortion correction performance - HCP

First four subjects from the Young Adult Human Connectome Project (Sotiropoulos et al., 2013) were used to visually evaluate the susceptibility-induced distortion correction performance between *TORTOISEV4* and the HCP pipeline. The provided HCP pipeline data underwent processing through a specialized HCP pipeline, which included the following steps: susceptibility distortion correction, inter- and intra-volume motion and eddy-currents distortion correction, outlier detection and replacement, gradient nonlinearity correction for diffusion sensitization and alignment to an anatomical image.

#### 2.3.3 Longitudinal validation - HCP test-retest

The first dataset used for quantitative validation as the HCP and HCP-Retest datasets, where the two scans of 40 subjects were reprocessed with *TORTOISEV4* and the HCP pipeline and output at the template space of the first scan’s T1W structural image. Diffusion tensors for all outputs were subsequently estimated, and fractional anisotropy (FA) and mean diffusivity (MD) maps were computed. For both pipelines, the difference images were computed in the subject-template space.

Instead of analyzing these subtraction maps individually in their native space, we opted to analyze them at the population level, on a common template space. To do this, we first created an unbiased template out of the actual diffusion tensors, T1W and T2W anatomical images from all subjects and all scans and saved the deformation fields mapping a subject’s scan onto this template. Using these transformations, we warped all the previously computed subtraction maps onto this template space. After this process, all these maps live in the same space, where they were averaged and quantified.

## 3 Results

### 3.1 Denoising and Gibbs ringing correction

**Fig. 3.**
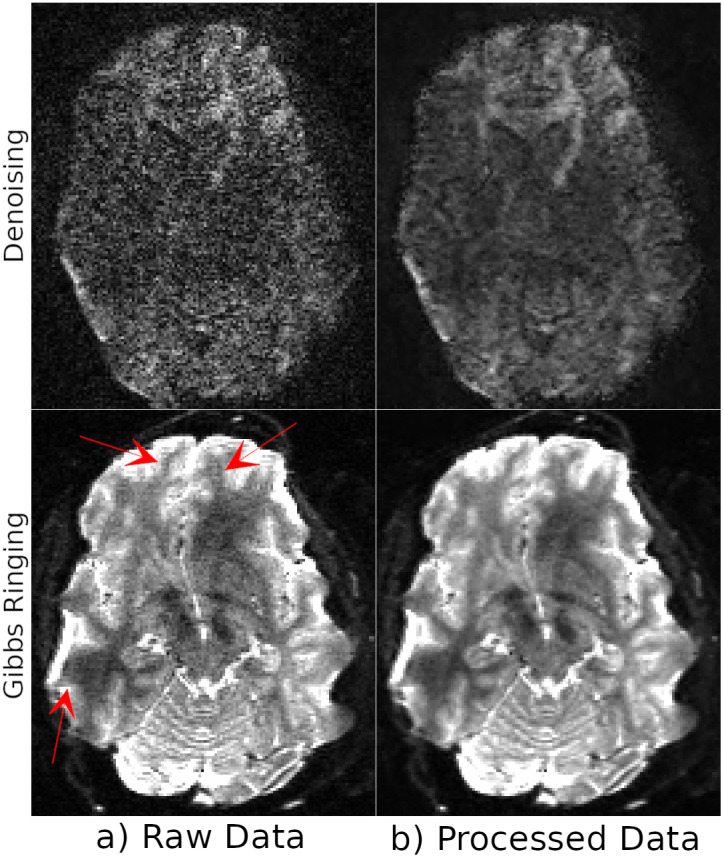
Denoising (top row) and Gibbs Ringing correction (bottom row) results from a single HCP subject with LR phase-encoding with the raw data on the left (Panel a) and processed data on the right (Panel b). The images displayed for denoising have a diffusion weighting of $b\;=\;3000s/mm^{2}$, and the Gibbs ringing images are non diffusion-weighted. Denoising significantly reduces the noise level, and structures that are barely visible in the raw images become distinct after processing. Red arrows point to the locations of visible Gibbs ringing artifacts. The correction procedure significantly reduces their effects but does not completely eliminate them.

Figure 3 displays the denoising and Gibbs ringing results of one HCP subject with LR phase encoding. As reported in their corresponding manuscripts (Kellner et al., 2016; Lee et al., 2021; Veraart et al., 2016) and validated by independent researchers, these processing strategies are very effective. The MP-PCA denoising methodology reduces the noise levels significantly on a heavily diffusion-weighted image, and structures that were originally barely distinguishable become distinctively clear after processing. In Figure 3, the red arrows in the bottom row point to the locations of observed Gibbs ringing artifacts. The $b\;=\;0s/mm^{2}$ image displayed at the bottom row (b) is an image that underwent denoising and Gibbs ringing correction. As can be observed, the ringing artifacts are significantly improved after processing but not fully eliminated in all regions. Additionally, we also observe a very slight smoothing effect with the performed Gibbs ringing correction; however, this effect is not significantly large to outweigh the benefits.

### 3.2 Within-volume motion correction and outlier detection & replacement

The correction of inter-volume motion and eddy-currents induced distortions have become quite robust and near-excellent over the past decade (J. L. Andersson & Sotiropoulos, 2016; Rohde et al., 2004). Therefore, in this section, we will provide sample results for intra-volume motion, that is, slice-to-volume motion, and outlier detection and replacement.

**Fig. 4.**
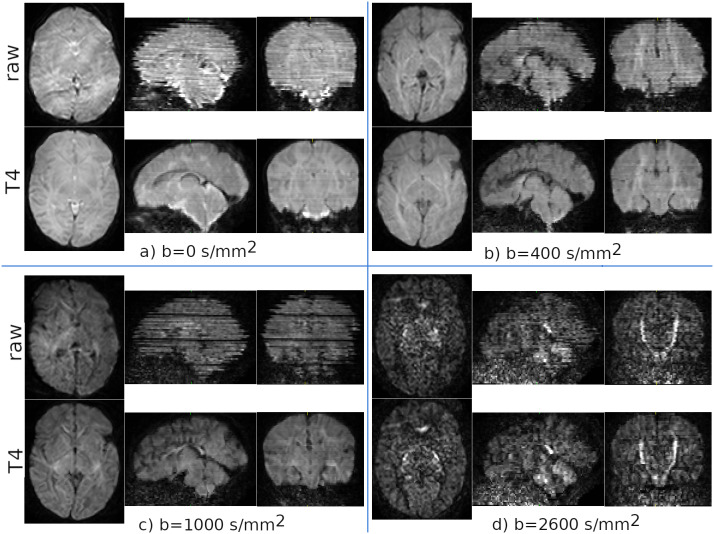
Intra-volume motion and outlier detection/replacement results from a single dHCP subject. Intra-volume motion and outlier detection/replacement processing quality of *TORTOISEV4* pipeline is illustrated in the second row given the artifacts that are present in the raw unprocessed data (first row). Panels (a)-(d) display sample processing results from individual shells. These images show that with such data suffering from significant motion-induced artifacts, *TORTOISEV4* processing is able to provide significantly improved data quality.

Figure 4 displays the *TORTOISEV4* processing results of a single dHCP subject, suffering from significant motion artifacts, in reference to the raw unprocessed dataset.

The panels in this figure display example cases of volumes from different shells. As can be observed especially from sagittal and coronal slices, *TORTOISEV4* significantly improves data quality considering the artifact levels present in all shells. This improvement is especially more evident for low b-value shells, where TORTOISE’s shell independent correction strategy did not suffer from the low number of volumes of these lower shells as the entire dataset is employed to synthesize the predicted signals. However, still, the correction quality is not perfect. For the b = 0 shell, remnants of signal fluctuations can still be observed between adjacent slices on sagittal and coronal views and even though ameliorated, lower signal level slices can still be observed on the $b\;=\;2600s/mm^{2}$ shell on the coronal and sagittal views.

**Fig. 5.**
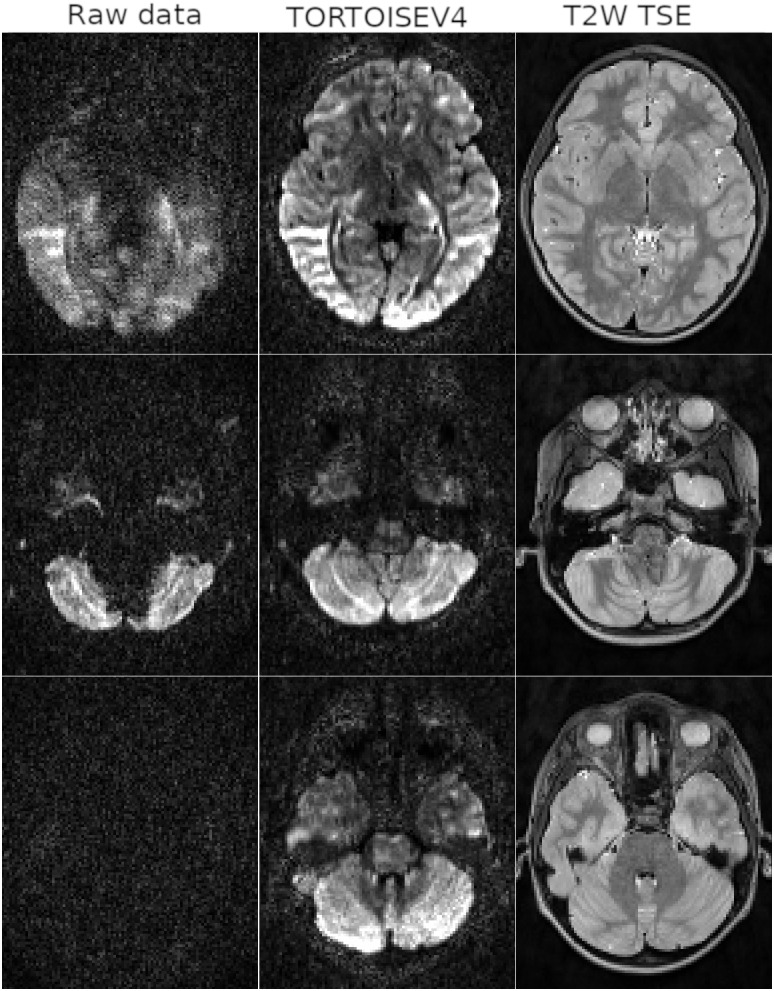
ABCD subject results. The first column displays the raw data, the next column the TORTOISEV4 processing outputs, and the last column displays the corresponding T2W anatomical image. *TORTOISEV4* results show that it can detect and correct most motion and cardiac pulsation induced signal dropouts.

Figure 5 displays the processing results of a single ABCD subject. Each row displays results from a different problematic volume and slice. For the first volume, the signal dropout affected the majority of the slice; however, the module was capable to detect the outliers and generate non-artifactual signals. The second volume suffers from typical cardiac pulsation induced signal dropouts, with most of the slice containing “good” data with a localized region suffering from drop-outs. For this volume, *TORTOISEV4* correctly replaced the voxels suffering from the drop-out and used the real data for the others. The third volume does not contain any signal from the brain at this slice level, therefore the entire slice was replaced with predictions. The reader should note that the correctness and accuracy of the replaced signals could not be validated for this dataset due to the lack of ground-truth. A representative but limited set of validations using simulated data with known ground-truths can be found in the Supplementary Materials SA3.

### 3.3 Susceptibility-induced distortion correction performance

**Fig. 6.**
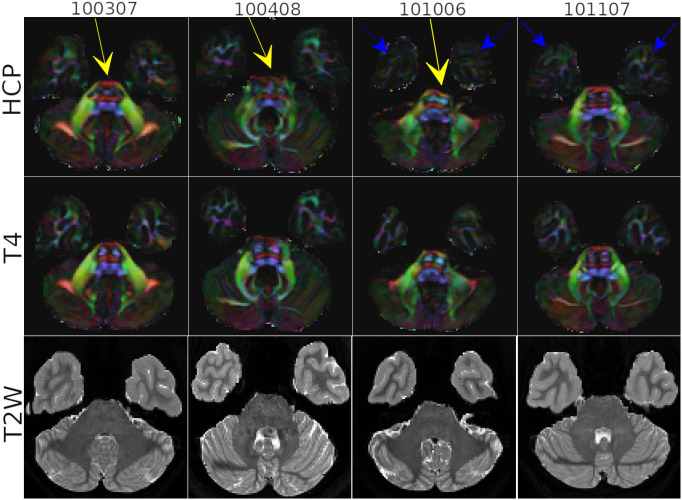
Directionally Encoded color maps four subjects of the HCP dataset. TORTOISE results are compared to the original HCP processing. Yellow arrows point to the Pons region where HCP processing was problematic and resulted in inaccurate representation of the cortical spinal tracts. Blue arrows indicate the differences in white matter anisotropy in temporal lobes with the two processing techniques. The T2W anatomical images are displayed at the bottom row for reference.

Figure 6 displays the Directionally Encoded color (DEC) maps from four subjects of the HCP dataset. The figure showcases a slice level that is typically significantly affected by susceptibility distortions. The reader should note that because the HCP and *TORTOISEV4* results originate from two separate pipelines, a small orientational difference exists between the two results originating from rigid registration to the anatomical image.

For the first three subjects in consideration, the white matter bundles inside the Pons are anatomically inaccurate with HCP processing (yellow arrows), as the cortical spinal tracts (CST) residing in both hemispheres are either non-existent (Subject# 100307) or present but with incorrect anatomy (Subject# 100408, 101006) due to an additional third tract in between the expected two lateral ones. *TORTOISEV4* processing, thanks to its use of tensorial information from both up and down data during the correction process, remedies this issue as for all the subjects two CST tracts are distinctly visible and anatomically more plausible. This issue was not observed with either pipeline for Subject# 101107. However, for this subject and subject# 101006, the white matter tracts in the temporal lobes were better delineated with the *TORTOISEV4* pipeline, exhibiting higher anisotropy (blue arrows).

**Fig. 7.**
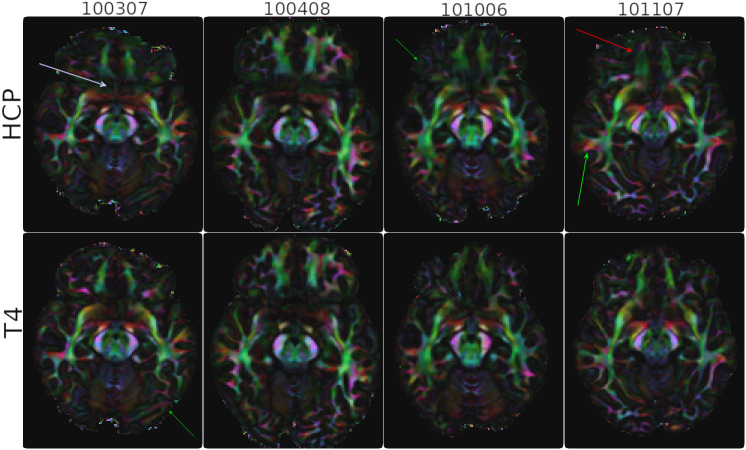
Directionally Encoded color maps four subjects of the HCP dataset at a mid-brain slice level. Red arrow points to the better delineation of the olfactory bulbs with T4. The other arrows point to the differences in sub-cortical white matter (green) and in olfactory bulb tracts (red, magenta).

Figure 7 displays the same maps at a mid-brain slice level. At this level, the differences between the two pipelines are still present but not as extreme as the Pons. Both pipelines produced similar results in the olfactory bulb region for the first three subjects. For subject# 101107, the bundles were better delineated with the *TORTOISEV4* pipeline (red arrow) and for subject# 100307, the HCP pipeline data was laterally more connected than T4 (lavender arrow), which was not anatomically accurate. Additional subcortical white matter differences were also observed as indicated by the green arrows.

**Fig. 8.**
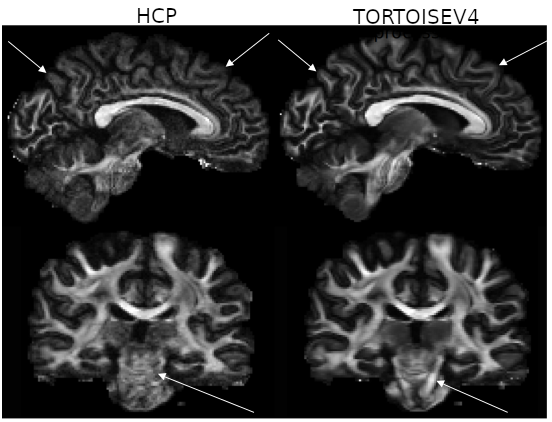
Sagittal and coronal views of an FA map of the first HCP subject. In *TORTOISEV4* version, the cortical spinal tracts (bottom arrows) are clearly visible and have a homogeneous directionality. Additionally, differences exist in the manifestation of several subcortical white matter pathways (top arrows).

Figure 8 displays the coronal and views of the FA maps of the first subject. The arrows point to the locations of the issues raised above, with *TORTOISEV4* generating anatomically more plausible images.

### 3.4 Gradient nonlinearities

**Fig. 9.**
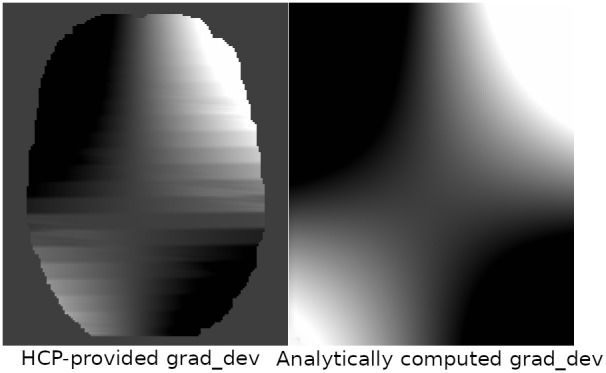
Map of the $L_{xy}$ component of gradient deviation tensors from a single HCP subject. The left image is provided by the HCP project and is based on numerical differentiation. The right image is provided by *TORTOISEV4* and is computed analytically from the spherical harmonics coefficients. Avoiding numerical differentiation eliminates the undesired striation effects present in the HCP version; therefore, it yields a more accurate estimation of the L tensor.

Figure 9 displays the $L_{xy}$ component map of the gradient deviation tensor L, which is an image that contains the 3 × 3 affine matrices that rotate and scale the diffusion gradients in each voxel. *TORTOISEV4* includes a mechanism to compute these tensors analytically from the provided manufacturer coefficients; therefore, it eliminates the need for numerical differentiation (which causes striation artifacts) and masking operations that were needed by the HCP pipeline, and therefore is more accurate.

**Fig. 10.**
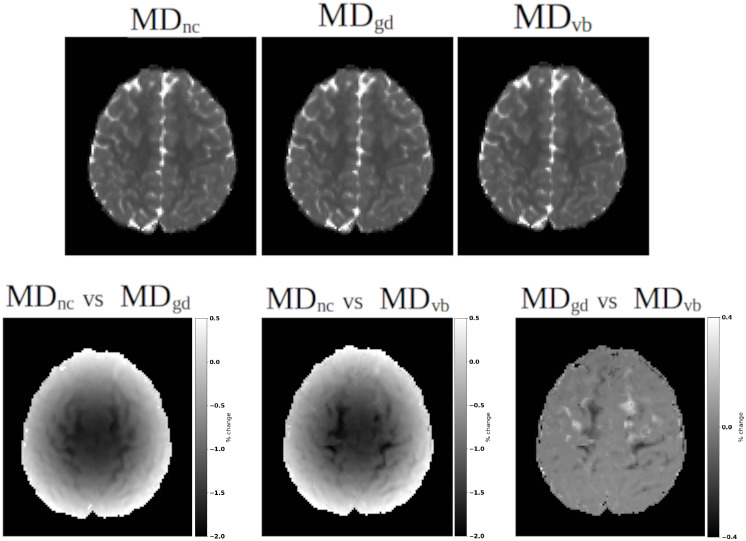
Effect of gradient nonlinearity correction on the Mean Diffusivity of an HCP subject. NC: no correction, GD: gradient deviation tensor based correction, VB: voxelwise B-matrix based correction, which also considers the effects of motion on nonlinearities. The top row displays the computed mean diffusivity maps, which visually appear near identical. The bottom row displays subtraction maps with a percentage based scaling. For the displayed slice level, correction of nonlinearity effects with GD yields a difference of up to 2% compared to no correction. With VB-based correction, this difference increases to 2.4%.

Figure 10 displays the comparisons of computed mean diffusivities when gradient nonlinearity correction is not applied (NC), is applied using the gradient deviation tensors (GD) and with voxelwise B-matrices (VB), which also considers the interaction between nonlinearities and motion. Performing gradient deviation tensor-based estimation yielded a difference of up to 2% in mean diffusivities for the displayed HCP subject. As expected, the differences were more pronounced at the very top (and bottom) of the brain near the cortex. Using voxelwise B-matrices in estimation yielded an additional 0.4% difference compared to gradient deviation tensor, suggesting a non-negligible effect of motion on these confounds.

### 3.5 Overall validation

**Fig. 11.**
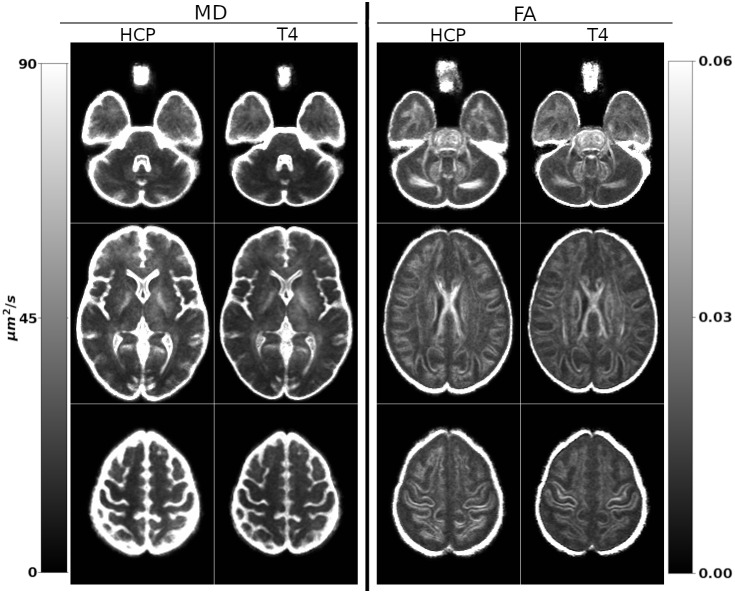
Average test-retest variability of FA and MD over the entire population for both pipelines. The window/level values are set identically within each modality. *TORTOISEV4* pipeline results in reduced variability (i.e., higher reproducibility) in the whole brain as indicated by the lower brightness levels. Especially in the body of the Corpus Callosum, the cortex, and the cerebellum, the difference is evident for both FA and MD.

In this section, we present the quantitative results of the entire pipeline validation using a test-retest approach as described in Section 2.2. Figure 11 displays the standard deviation maps of HCP test-retest data, warped onto a template space and averaged over the population, that is, population-level test-retest variability maps for MD and FA. The variability maps are displayed at three slice levels. At first visual inspection, the overall variability is reduced by the *TORTOISEV4* pipeline, as indicated by the decreased brightness levels in all the images. This is especially evident at the lower brain level (first row) and the top brain level (third row), which implies that on average, the HCP test and retest data are more similar to each other after *TORTOISEV4* processing. Additionally, The HCP pipeline resulted in large variability in the cerebellum white matter regions, which was reduced with T4 processing. Similar behavior was also observed in the body of the Corpus Callosum.

Table 2 quantifies and summarizes the standard deviations of the population-level maps for several ROIs. *TORTOISEV4* improved test-retest quantitative reproducibility nearly for all ROIs, for both metrics, as initially observed from the deviation maps. The improvements for mean diffusivity were up to 19%, which was achieved in the whole brain. FA reproducibility was more similar between the two pipelines, where *TORTOISEV4* produced better results in the cerebellum (up to 25%), whole white matter, and the whole brain. In the internal capsule and temporal lobes, the HCP pipeline produced more reproducible results. As can actually be observed in the maps of Figure 11, in the temporal lobes, *TORTOISEV4* results in lower FA variability within the white matter; however, in gray matter, the HCP pipeline is more stable.

**Table 2.** Average ROI values from the population-level standard deviation maps.

MD ($\mu m^{2}/s$ )			FA	
	HCP	T4	HCP	T4
Cerebellum	28.04	23.79	0.027	0.020
Temporal lobes	36.03	32.25	0.024	0.025
Body of CC	26.60	23.02	0.027	0.022
Internal Capsule	25.00	21.02	0.029	0.033
WM (FA ≥ 0.3)	21.18	19.82	0.022	0.021
Whole Brain	35.48	28.79	0.022	0.019

Lower values indicate more similarity between the test and retest data over the population. *TORTOISEV4* results in more reproducible results between the test and retest data for all the ROIs for both metrics, where the only exceptions were the temporal lobes and internal capsules for FA. Diffusivity units are in $\mu m^{2}/s$, which are $10^{3}$ larger than the commonly used $\mu m^{2}/ms$.

## 4 Discussion

Inaccuracy and low reproducibility of diffusion-derived metrics has been a major obstacle to the adoption of quantitative diffusion MRI (dMRI) in the clinical setting. One of the sources of these imperfections is the artifacts and distortions that are prominent in Echo Planar Imaging (EPI). Most of these distortions and artifacts can be improved upon or even virtually eliminated with image processing techniques. Therefore, dMRI preprocessing plays a crucial role in ensuring the accuracy and reliability of subsequent analyses.

Image processing is a fast evolving field, especially due to recent advancements in machine learning, which has a direct impact on dMRI preprocessing. Novel advancements for each artifact or distortion type involved in dMRI are proposed continuously. Despite the ever increasing availability of tools and techniques for dMRI preprocessing, running an “optimized” preprocessing pipeline can be a challenging task even for experts as the data flow from acquisition to processing to modeling becomes more integrated. Additionally, with more sophisticated acquisition schemes beyond the standard Stejskal-Tanner (Stejskal & Tanner, 1965) Pulse-Gradient-Spin-Echo are becoming increasingly more common, the optimal choice of preprocessing steps and pipeline choice may highly depend on the data, and become quickly outdated with new tools becoming available.

In this work, we have presented the new *TORTOISE* preprocessing pipeline, *TORTOISEV4*, which was developed with a design philosophy that caters to most dMRI preprocessing needs: richness of features, ease-of-use, and modularity for inter-operability with other packages. *TORTOISEV4*’s current module set can be considered as a snapshot of most commonly needed/used features in dMRI preprocessing at the time of development. With the ever-evolving preprocessing needs, the modular structure of *TORTOISE* will enable a faster development cycle while making it easier for both the developers and the community to integrate novel features to the pipeline. Conversely, *TORTOISE* modules can be used by researchers and clinicians independently without invoking the full pipeline or by the developers who can seamlessly integrate *TORTOISE* modules into their own pipelines. As an example of this case, TORTOISE’s susceptibility distortion correction module *DRBUDDI*, gradient nonlinearity correction modules, and the diffusion tensor/MAPMRI estimation modules have already been integrated into QSIPREP (Cieslak et al., 2021) with the intra-volume motion and outlier detection/replacement modules’ integration being underway.

Even though *TORTOISEV4* contains a module for “most” dMRI preprocessing steps, it still is not a complete set at the time of release and lacks functionality for several operations or data types.

- One of these functionalities is the re-estimation of susceptibility per volume, as pioneered by the *FSL* package. Even though *TORTOISEV4* rotates the estimated fieldmap based on motion parameters, it still assumes a constant underlying susceptibility map. However, for matching DWI sets between the blip-up and blip-down datasets, it supports “per-volume” estimation of the susceptibility maps within its GPU-based implementation to keep the computation time within an acceptable range.
- Another feature that was not released at the time of writing is about detecting and correcting for the EPI ghost artifacts. A future work will describe *TORTOISEV4*’s approach to ghost correction, which makes use of dMRI data acquired with all four phase-encoding directions, that is, Anterior-Posterior, Posterior-Anterior, Right-Left, and Left-Right phase-encoding.
- *TORTOISEV4* has been designed for linearly encoded, constant echo time dMRI datasets. However, with the recent resurgence of multi-dimensional acquisitions including planar/spherical encoding schemes with multi TE/TR, the community will soon need publicly available and generalizable dMRI pre-processing pipelines. Such data mostly affect the signal estimation and re-synthesis components of pipelines. Currently, *TORTOISEV4* employs signal resynthesis based on the MAPMRI model for slice-to-volume registration and outlier detection/replacement functionalities; therefore, enabling these modules for such data is currently not advised. However, extensions to our signal estimation routines are being developed to account for multi-dimensional datasets. These extensions will be available in a future sub-release after testing.

During the development of *TORTOISEV4*, several large single or multi-site dMRI datasets have been completely reprocessed to validate the accuracy, performance, and reproducibility of *TORTOISE* outputs. These publicly available datasets included the young Adult Human Connectome project dataset (HCP), the developing Human Connectome Project (dHCP) dataset, and a large number of subjects from the Adolescent Brain Cognitive Development (ABCD) project. We aim to make most of these reprocessed datasets publicly available. The entire HCP1200 dataset, along with its gradient nonlinearity corrected version GDFA, will be made publicly available once a data server/host is decided upon. The ABCD project will already include several *TORTOISEV4* modules in its processing for its next official release.

The overall *TORTOISEV4* pipeline and one of its modules had not been systematically validated before this work using these datasets. Due to a lack of ground-truth, how to assess whether one acquisition scheme or one preprocessing scheme is better than another one has always been an outstanding question in the field. Simulations help in this regard but quantitatively validating a tool is still a challenge. In this work, our approach was to assess longitudinal variability of test-retest datasets assuming biological changes were minimal due to the short time difference between the scans used in this work. We believe that this approach is the most promising quantitative validation strategy for such a question but it is not without caveats. One caveat is its sensitivity to smoothing. An extreme example would be a processing pipeline that always generates blank images regardless of the input. Such a pipeline would always achieve perfect longitudinal reproducibility (variance), even though the accuracy (mean) naturally suffers. Additionally, when longitudinal variability is analyzed on dMRI-derived maps, which are generated using the entire dMRI dataset, different types of imperfect processing, such as failed intra-volume motion correction for different volumes, might lead to similar smoothing levels between the test and retest versions of the datasets, which this approach might not be able to pinpoint.

The *TORTOISEV4* module that still needs a systematic and thorough validation is the intra-volume motion and outlier detection and replacement algorithm. Due to space constraints, we decided to focus on this validation in a separate research paper that is part of ongoing work. We hope this validation will involve help from community members and other pipelines’ developers to systematically but fairly assess all similar toolkits’ performances.

## 5 Conclusions

*TORTOISE* is one of the oldest diffusion MRI preprocessing pipelines and has its roots in the very first diffusion tensor estimation implementation. However, until mid-late 2010s, it was not publicly available. Even after its public release, its closed source format and multi-step processes, requiring manual intervention, made it less user friendly. *TORTOISEV4* aims to improve upon the previous versions’ unideal characteristics while providing a state-of-the-art feature set and improved performance to cater to the community’s rising dMRI processing needs.

## Supplementary Material

Supplementary Material

## Data Availability

All the datasets used for this work are already publicly available. The source code and the compiled binaries of *TORTOISEV4* can be found at: https://github.com/QMICodeBase/TORTOISEV4.
