# Supplementary material for: TORTOISEV4: Reimagining the NIH diffusion MRI processing pipeline
Source: Imaging Neurosci (Camb). 2025 Dec 9;3:IMAG.a.948. doi: 10.1162/IMAG.a.948 (PMC12690295; doi:10.1162/IMAG.a.948)
Supplement: Supplementary Material [file IMAG.a.948_supp.pdf]

TORTOISEV4: ReImagining the NIH Diffusion MRI Processing Pipeline - Supplementary Materials

M. Okan Irfanoglu,[1*] Amritha Nayak,[1] Paul Taylor,[2] Anh Thai,[1,3] Carlo Pierpaoli,[1]

[1]Quantitative Medical Imaging Laboratory, NIBIB, National Institutes of Health, Bethesda, 20892, MD, USA

[2]Scientific and Statistical Computing Core, NIMH, National Institutes of Health, Bethesda, 20892, MD, USA

[3]Electrical Engineering Department, Catholic University of America, Washington DC, 20064, USA

[*]Correspondence: irfanoglumo@nih.gov

August 28, 2025

A Supplementary Materials

A.1 Module Implementation and Default Settings

In this section, the implementation details and default settigns will be described for each TORTOISEV4 module. The subsections' titles will include the module name (i.e. executable name) in italic. The runtimes were measured on a single HCP subject data, including both phase encoding directions, on a 64 core AMD Threadripper CPU equipped with an NVIDIA RTX 4090.

A.1.1 Denoising - *DWIDenoise*

MP-PCA has been re-implemented in *TORTOISEV4* based on MRTRIX3's (Tournier et al., 2019) EIGEN-based implementation but with further improvements on boundary conditions. The module takes a single parameter defining the kernel size, which in the main *TORTOISEProcess* pipeline is automatically computed based on the total number of volumes as suggested by the original paper (Veraart et al., 2016).

- Method: MP-PCA
- Default kernel diameter: $max\left(9, round(Nvolumes^{1/3})\right)$
- Runtime: 14mins

A.1.2 Gibbs Ringing - *Gibbs*

TORTOISEV4's Gibbs ringing correction uses the original authors' implementation of the local subvoxel shift algorithm (Kellner et al., 2016), where Lee et al. (2021) extensions were implemented in-house. The parameters to the module are the k-space coverage of the data, the phase-encoding direction (which are both automatically read from the corresponding JSON files in the main pipeline), $minW$ (starting point of the oscillation search window), $maxW$ (end point of oscillation search window) and NSH (sampling density of the $2D$ weighting filter.

- Method: Subvoxel-shift or RPG
- k-space coverage: Read from JSON file or input as parameter
- minW=1
- maxW=3
- NSH=25
- Runtime: 2mins

A.1.3 Motion and Eddy-Currents Distortions - *DIFFPREP*

When the slice-to-volume submodule is enabled, *DIFFPREP* is computationally quite expensive, due to a larger number of registrations and non-grid based forward interpolations, therefore, it is heavily parallelized and is designed to take advantage of multi-core CPU architectures or (multi) CUDA-capable GPU devices. For instance, for a Human Connectome Project subject with 288×2 volumes (RL and LR phase encoding), the procedure takes about 45 minutes when slice-to-volume is disabled and about six hours when enabled, on a 64-core AMD EPYC CPU architecture equipped with a single NVIDIA RTX 4090 GPU. The "system_settings.json" file in the distribution defines what percentage of available CPU cores *DIFFPREP* can employ, to be compatible with several cluster runtime limitations.

DIFFPREP can take a large number of parameters as input to define its run-time behaviour. Here, we will report only their default values and not all the possible options, which the executable helps and the documentation can provide.

- best $b = 0$ image id: Automatic

- is human brain?: Yes
- center: isocenter of the scanner from the NIFTI file
- correction type: Inter-volume motion and eddy-currents with a quadratic transformation
- slice-to-volume: off
- outlier detection: off
- Percentage of outliers for a volume to be considered unusable: 50%
- Probability threshold for labeling a slice an outlier: 0.025
- Outlier detection behaviour: Neither conservative nor aggressive.
- Number of iterations: 3
- Runtime: 345mins

A.1.4 Signal Drift - *DWIDrift*

Signal drift is computed from temporarily registered $b = 0\ s/mm^2$ images from *DIFFPREP* within a brain mask. The mask is automatically computed using *FSL*'s BET2, however, for non-human data, the users have the option to provide and externally computed binary mask. The module also checks whether the distribution of the $b = 0$ images is sufficiently dispersed over the acquisition and automatically rejects to perform the correction if the data is unsuitable (such as all $b = 0$ images at the beginning of the acquisition).

- Regression method: Linear
- Rejection to apply when: $N_{volumes} < 30$
- Rejection to apply when: $N_{b=0} < 4$
- Rejection to apply when: $\max\left(\{volume\_id(b = 0^{i+1}) - volume\_id(b = 0^{i})\}\right) < 1.2N_{volumes}/N_{b=0}$
- Runtime: 1sec

A.1.5 Susceptibility Distortions - *DRBUDDI*

DRBUDDI supports both multi-threaded CPU-based computations and CUDA-based GPU computations. For an HCP subject, the CPU based correction takes about 1.5 hours and the GPU version takes about 3 minutes. Due to this significant difference in computational times, a simpler gradient-descent based optimization strategy was implemented for the CPU version and a more expensive Conjugate-Gradient based optimizer with line-search for the GPU version. As the conjugate gradient based scheme can be hypothesized to generate better results thanks to its line search, the GPU and CPU version results will not be identical, with the GPU version expected to yield slightly improved correction quality.

The following default settings are used by *DRBUDDI* :

- Gradwarp application to $b = 0$ images: ON
- Used $b = 0$ images: Estimated from tensor fitting to whole dataset
- Use FA images: On
- Rigid registration between up/down/structural images: On
- Transformation type: SyN
- Runtime: 3mins

DRBUDDI has 28 stages. The details of each stage are described in Table 1. In this table, "restrict" refers to a parameter that enforces all deformations to be along the phase encoding direction and "constrain" enforces up-down deformation anti-symmetry.

Stages	Metrics	Img smoothing/Downsampling	Step size	Field smoothing	Restrict/Constrain
1	MSJac	4.5/8	0.35	13.5/0.05	True/True
2	MSJac	4/8	0.25	11.5/0.05	True/True
3	MSJac	3.5/8	0.15	9.5/0.05	True/True
4	MSJac	3./8	0.3	7.5/0.05	True/True
5	MSJac	2.5/8	0.05	5.5/0.05	True/True
6	MSJac,CCJacS	2./8	0.2	4.5/0.05	True/True
7	MSJac,CCSK	4/8	0.5	5/0.05	True/True
8	MSJac,CC,CCJacS	4/6	0.35	11.5/0.05	True/True
9	MSJac,CC,CCJacS	3.5/6	0.3	9.5/0.05	True/True
10	MSJac,CC,CCJacS	3./6	0.4	7.5/0.05	True/True
11	MSJac,CC,CCJacS	2.5/6	0.2	6.5/0.05	True/True
12	MSJac,CC,CCJacS	2/6	0.1	5.5/0.05	True/True
13	MSJac,CC,CCJacS	2/6	0.15	4.5/0.05	True/True
14	MSJac,CC,CCJacSK	2/6	0.75	3.5/0.05	True/True
15	MSJac,CC,CCJacS,CCSK	3/4	0.75	9.5/0.1	True/True
16	MSJac,CC,CCJacS,CCSK	4/4	0.75	7.5/0.05	True/True
17	MSJac,CC,CCJacS,CCSK	3.5/4	0.5	5.5/0.05	True/True
18	MSJac,CC,CCJacS,CCSK	3/4	0.35	4.5/0.05	True/True
19	MSJac,CC,CCJacS,CCSK	3/4	0.25	3.5/0.05	True/True
20	MSJac,CC,CCJacS,CCSK	2.5/4	0.2	3.0/0.05	True/True
21	MSJac,CC,CCSK	2/4	1.	5.0/0.1	True/True
22	MSJac,CC,CCJacS,CCSK	2/2	1.0	7.5/0.05	True/True
23	MSJac,CC,CCJacS,CCSK	1/2	0.85	5.5/0.05	True/True
24	MSJac,CC,CCJacS,CCSK	0.5/2	0.75	4.5/0.05	True/True
25	MSJac,CC,CCJacS,CCSK	1/1	1.5	7.5/0.05	True/True
26	MSJac,CC,CCJacS,CCSK	0.25/1	1.	5.5/0.	True/True
27	MSJac,CC,CCJacS,CCSK	0/1	0.9	4.5/0.	False/False
28	CC,CCJacS,CCSK	0/1	0.1	3./0.	False/False

Table 1: *DRBUDDI* stage settings. Smoothing factors are standard deviations of Gaussian kernels in voxels.

In this table, the only information that is not provided is the default weights of the metrics at each stage. These weights are not constant over stages and were selected after extensive experimentation

with different types of datasets. The principle while setting these weights was not to get the optimum result for one type of dataset but to get "better than acceptable" result for most datasets. To be more specific, the MSJac metric, i.e. the traditional Mean Squares difference with Jacobian signal manipulation, is aassigned a larger weight in the earlier stages of processing for downsampling factors $8x6x4x$, after which the structural image based metrics have their weights increase with each stage to full importance weighting in the last stage. The tensors based metrics have a spatially varying weight that is automatically decided based on the current voxels FA.

The user still has the option to overwrite these settings and change any metric or its corresponding weight.

A.2 *DRBUDDI* Metrics and Gradients

DRBUDDI employs several similarity metrics to simultaneously achieve several purposes. The definition and the gradients of the CC metric, which uses FA images, is identical to the original description by Avants et al. (2008). The $CCSK$ metric's definitions can be found in the original *DRBUDDI* paper (Irfanoglu et al., 2015). *TORTOISEV4* introduced the $MSJac$ and $CCJacS$ metrics. The definitions of the $CCJacS$ metric is derived from a combination of the $CCSK$ and $MSJac$ metrics, therefore in this session, we will present the new formulation of the $MSJac$ metric only.

Let I_{up} and I_{down} be the original distorted images, I'_{up} and I'_{down} be their geometrically transformed versions to the middle timepoint, i.e. the undistorted space, with corresponding fields ϕ_{up} and ϕ_{down} as:

$$I'_{up}(\mathbf{x}) = I_{up}(\phi_{up}(\mathbf{x})) \tag{1}$$

$$I'_{down}(\mathbf{x}) = I_{down}(\phi_{down}(\mathbf{x})) \tag{2}$$

The total similarity metric is defined as:

$$\xi = w_{MSJac}\xi_{MSJac} + w_{CC}\xi_{CC} + w_{CCJacS}\xi_{CCJacs} + w_{CCSK}\xi_{CCSK}$$

with weights w varying at each stage but summing up to 1 for all employed metrics.

A.2.1 MSJac

The MSJac metric computes the similarity of Jacobian multiplied $b = 0$ images as:

$$\xi_{MSJac} = \frac{1}{N}\sum_{\mathbf{x}\in\Omega}\left(I'_{up}(\mathbf{x})||\mathcal{J}(\phi_{up}(\mathbf{x}))|| - I'_{down}(\mathbf{x})||\mathcal{J}(\phi_{down}(\mathbf{x}))||\right)^2$$

where $\mathcal{J}$ is the operator computing the Jacobian matrix of a deformation field and $||.||$ is the determinant operator.

Let us denote the residual term inside the parantheses as $r(\mathbf{x})$. Then, the gradient descent direction for the up field can be described as:

$$\frac{\partial \xi_{MSJac}}{\partial \phi_{up}}(\mathbf{x}) = \frac{2}{N}\sum_{\mathbf{x}\in\Omega} r(\mathbf{x})\frac{\partial\left(I'_{up}(\mathbf{x})||\mathcal{J}(\phi_{up}(\mathbf{x}))||\right)}{\partial \phi_{up}(\mathbf{x})}$$

The second gradient term can be expressed as:

$$\frac{\partial\left(I'_{up}(\mathbf{x})||\mathcal{J}(\phi_{up}(\mathbf{x}))||\right)}{\partial \phi_{up}(\mathbf{x})} = \frac{\partial I'_{up}(\mathbf{x})}{\partial \phi_{up}(\mathbf{x})}||\mathcal{J}(\phi_{up}(\mathbf{x}))|| + I'_{up}(\mathbf{x})\frac{\partial||\mathcal{J}(\phi_{up}(\mathbf{x}))||}{\partial \phi_{up}(\mathbf{x})} \tag{3}$$

As presented by Avants et al. (2008), the gradient term $\frac{\partial I'_{up}(\mathbf{x})}{\partial \phi_{up}}$ is simply the image gradients of the warped image $\nabla I'_{up}$. The gradient of the Jacobian term is however more complicated: as the Jacobian matrix of a displacement field at voxel $\mathbf{x}$ does not change w.r.t the displacement at that voxel, the Jacobian gradient term is by definition 0 at voxel $\mathbf{x}$. However, neighboring voxels along the phase-encoding direction do contribute to it. Therefore, unlike SyN formulation (Avants et al., 2008), the neighboring voxels need to be considered and the gradients need to be reformulated. Assuming the Jacobians are computed from a 3×3 window:

$$\frac{\partial \xi_{MSJac}}{\partial \phi_{up}(\mathbf{x})} = \frac{\partial \xi_{MSJac}(\mathbf{x})}{\partial \phi_{up}(\mathbf{x})} + \frac{\partial \xi_{MSJac}(\mathbf{x}+\mathbf{1})}{\partial \phi_{up}(\mathbf{x})} + \frac{\partial \xi_{MSJac}(\mathbf{x}-\mathbf{1})}{\partial \phi_{up}(\mathbf{x})}$$

As stated above, the first term $\frac{\partial \xi_{MSJac}(\mathbf{x})}{\partial \phi_{up}(\mathbf{x})}$ is simply $\frac{2}{N} r(\mathbf{x})\nabla I'_{up}||\mathcal{J}(\phi_{up}(\mathbf{x}))||$. The second term:

$$\frac{\partial \xi_{MSJac}(\mathbf{x}+\mathbf{1})}{\partial \phi_{up}(\mathbf{x})} = \frac{2}{N}\sum_{x\in\Omega} r(\mathbf{x}+\mathbf{1})\frac{\partial\left(I'_{up}(\mathbf{x}+\mathbf{1})||\mathcal{J}(\phi_{up}(\mathbf{x}+\mathbf{1}))||\right)}{\partial \phi_{up}(\mathbf{x})}$$

with

$$\frac{\partial\left(I'_{up}(\mathbf{x}+\mathbf{1})||\mathcal{J}(\phi_{up}(\mathbf{x}+\mathbf{1}))||\right)}{\partial\phi_{up}(\mathbf{x})} = \frac{\partial I'_{up}(\mathbf{x}+\mathbf{1})}{\partial\phi_{up}(\mathbf{x})}||\mathcal{J}(\phi_{up}(\mathbf{x}+\mathbf{1}))||+ I'_{up}(\mathbf{x}+\mathbf{1})\frac{\partial||\mathcal{J}(\phi_{up}(\mathbf{x}+\mathbf{1}))||}{\partial\phi_{up}(\mathbf{x})} \quad (4)$$

By chain rule,

$$\frac{\partial I'_{up}(\mathbf{x}+\mathbf{1})}{\partial\phi_{up}(\mathbf{x})} = \frac{\partial I'_{up}(\mathbf{x}+\mathbf{1})}{\partial\phi_{up}(\mathbf{x}+\mathbf{1})}\frac{\partial\phi_{up}(\mathbf{x}+\mathbf{1})}{\partial\phi_{up}(\mathbf{x})}$$

with $\frac{\partial I'_{up}(\mathbf{x}+\mathbf{1})}{\partial\phi_{up}(\mathbf{x}+\mathbf{1})}$ as $\nabla I'_{up}(\mathbf{x}+\mathbf{1})$. The term $\frac{\partial\phi_{up}(\mathbf{x}+\mathbf{1})}{\partial\phi_{up}(\mathbf{x})}$ should normally be zero as the displacement vector at a voxel is independently computed. However, as a regularization step, we smooth our deformation fields with a Gaussian kernel, therefore a voxel indeed affects its neighbors. Therefore, at each stage, the term $\frac{\partial\phi_{up}(\mathbf{x}+\mathbf{1})}{\partial\phi_{up}(\mathbf{x})}$ is a small constant number computed from the Gaussian kernel size.

The term $\frac{\partial||\mathcal{J}(\phi_{up}(\mathbf{x}+\mathbf{1}))||}{\partial\phi_{up}(\mathbf{x})}$ can be directly extracted from the discrete computation of the Jacobian. Assuming the phase-encoding direction is vertical, the term:

$$\mathcal{J}_{11}(\phi_{up}(\mathbf{x}+\mathbf{1})) = \frac{\phi^y_{up}(y+2)-\phi^y_{up}(y)}{2\Delta y}$$

therefore, the derivative is simply $\frac{-1}{2\Delta y}$ with Δy being the voxel spacing along the y direction. For voxel $\mathbf{x}-\mathbf{1}$, this term will be positive.

A.3 Intra-volume Slice-to-volume Motion Simulation

The performance of the intra-volume motion and outlier detection module was not systematically tested and contrasted to other pipelines in the main manuscript. For completeness, we performed simulations to validate its accuracy. For this purpose, an HCP subject data with little to no motion was selected and 100 volumes were extracted from the motion corrected version of this data. About one third of the volumes were randomly selected to be contaminated by motion. Within these selected volumes, up to four slice multi-band groups (up to 12 slices) were replaced by artificially rotated and translated synthetic slices computed from the MAPMRI model. Rotations were restricted to be up to 45 degrees. For the corrupted slices, the ground truth rotations and the estimated ones were compared.

Figure 1 displays three adjacent axial slices in a corrupted volume along with a sagittal slice at the top row. The rotation was about 30° along the x-axis for this example. The images in the bottom row

display the processed data. For this illustrative case, *TORTOISEV4* was able to correctly correct for the artificially introduced motion.

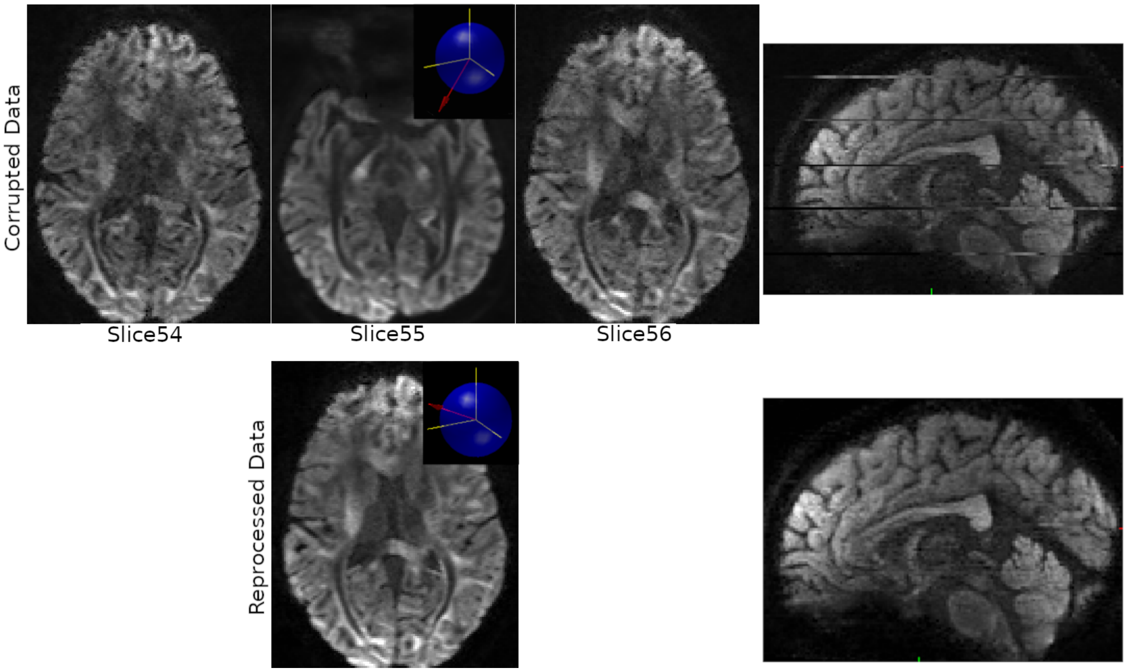

Figure 1: An HCP subject data artificially corrupted with motion. For this example case, the motion affected two slice groups (6 slices) and was about 30° along the x-axis. The processing was able to correctly capture the rotation and translation levels. The blue circles and arrows indicate the effective diffusion gradients before and after processing.

Figure 2 displays the error in estimated rotations compared to the ground truth for corrupted slices per volume. For each volume, the difference between ground truth rotations and estimated rotations are averaged over affected slices and plotted for each volume. As can be seen from the bar chart, the maximum error in prediction is about 2.5^o, which is considerably smaller than the introduced 45 degree rotation. Still, the correction is not perfect as 2.5^o might be considered to be non-negligible. We would also like to state that in our experiments, this level of corruption (30% of volumes, 12 slices per volume, up to 45 degree rotation) turned out to be the upper limit in terms of performance, after which the correction quality started to diminish rapidly with the quantitative rotation errors rising.

A.4 Final Data Generation Interpolation Strategy Slice-to-Volume Registration

When slice-to-volume registration is not enabled, TORTOISEV4 performs its interpolation during the final data generation phase using the traditional cubic B-Splines as all the transformations are combined into

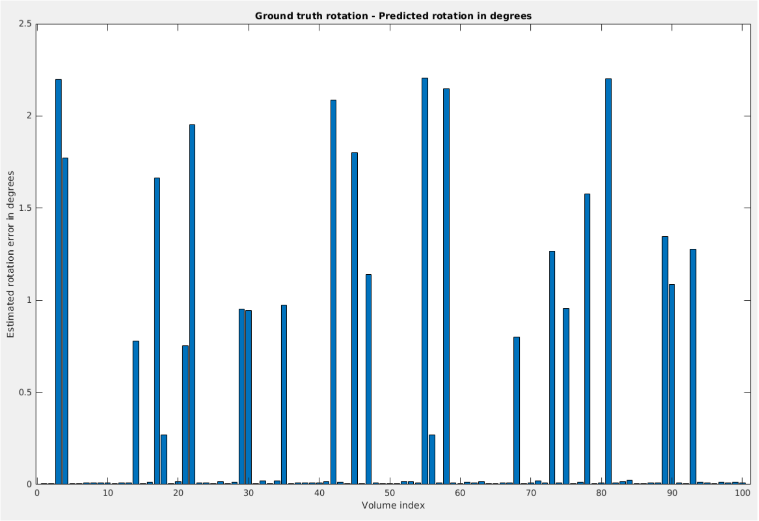

Figure 2: Quantitative errors in rotation using the simulated data. The bar chart displays the average error between the ground truth rotation and the estimated one per volume. Even though the errors are relatively small, i.e. less than 2.5 degrees, the correction is not perfect with such large data corruption levels.

a single backward transformation prior to interpolation. However, when intra-volume motion correction is enabled, the slice-to-volume transformations are forward, which necessitates a different interpolation strategy, especially considering missing data due to large motion. The interpolation method employed by TORTOISE is described in the flowchart depicted below:

Prior to final data generation with interpolation, all transformations are converted to deformation fields, including slice-to-volume. Subsequently, for each voxel $\mathbf{X}$ on the final subject template space, TORTOISE first checks whether the s2v field is locally diffeomorphic by first backward transforming $\mathbf{X}$ with the inverse s2v field, and then forward transforming it with the actual s2v transformation to get to point $mathbfX'$. If $||\mathbf{X} - \mathbf{X}'|| < \epsilon$, the field is considered diffeomorphic and a traditional backward interpolation is used. If not, all points of the image are forward transformed on to the final space. They form an irregular point cloud instead of an image grid after this operation. Sixteen closest neighbors of $\mathbf{X}$ are searched for using a kd-tree spatial structure. If $\mathbf{X}$ lies inside the convex hull of these neighbors, a Gaussian Radian Basis (RBF) interpolation is employed if the resulting RBF matrix is well conditioned. If $\mathbf{X}$ is outside the convex hull or the RBF interpolant is not well conditioned, TORTOISEV4 checks whether there is even sufficient data for interpolation. IF there is, an inverse distance weighted interpolation is used with a distance power of 8, if there isn't, the algortihm considers this case as "missing data" either due to large motion or artifacts and instead of interpolation, it uses the predicted signal from the MAPMRI model to generate the final signal value.

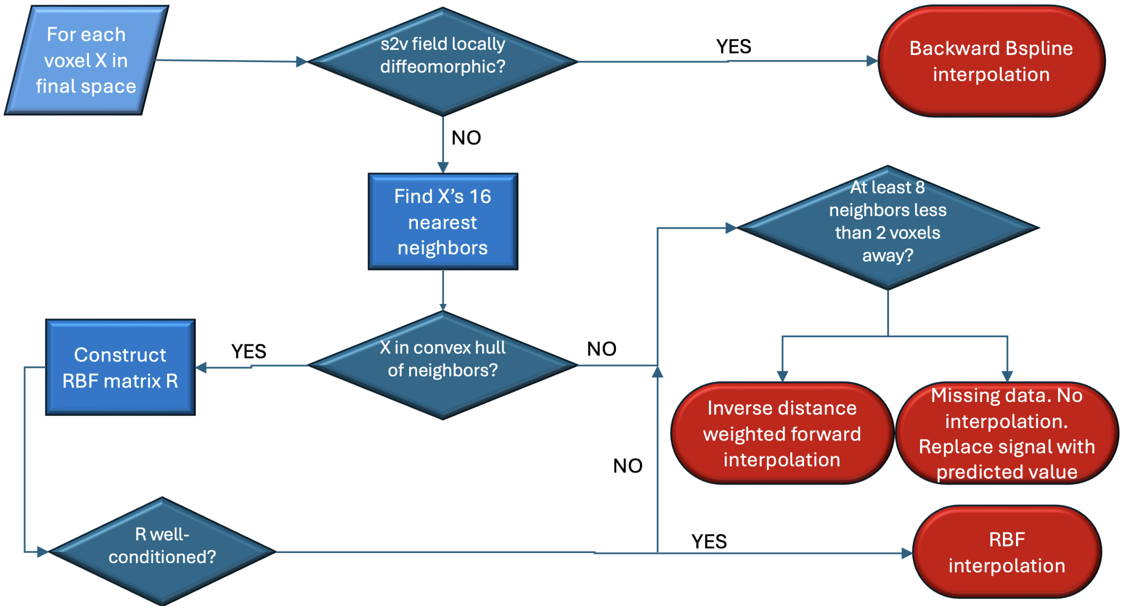

Figure 3: Interpolation strategy for data processed with slice-to-volume registration enabled.

References

Avants, B., Epstein, C., Grossman, M., & Gee, J. (2008). Symmetric diffeomorphic image registration with cross-correlation: Evaluating automated labeling of elderly and neurodegenerative brain. *Medical Image Analysis*, *12*(1), 26–41.

Irfanoglu, M. O., Modi, P., Nayak, A., Hutchinson, E. B., Sarlls, J., & Pierpaoli, C. (2015). DR-BUDDI: (Diffeomorphic registration for blip-up blip-down diffusion imaging) method for correcting echo planar imaging distortions. *Neuroimage*, *106*, 284–289.

Kellner, E., Dhital, B., Kiselev, V. G., & Reisert, M. (2016). Gibbs-ringing artifact removal based on local subvoxel-shifts. *Magnetic Resonance in Medicine*, *76*(5), 1574–1581.

Lee, H.-H., Novikov, D. S., & Fieremans, E. (2021). Removal of partial fourier-induced gibbs (RPG) ringing artifacts in MRI. *Magnetic Resonance in Medicine*, *86*(5), 2733–2750. https://doi.org/https://doi.org/10.1002/mrm.28830

Tournier, J.-D., Smith, R., Raffelt, D., Tabbara, R., Dhollander, T., Pietsch, M., Christiaens, D., Jeurissen, B., Yeh, C.-H., & Connelly, A. (2019). Mrtrix3: A fast, flexible and open software framework for medical image processing and visualisation. *NeuroImage*, *202*, 116137. https://doi.org/https://doi.org/10.1016/j.neuroimage.2019.116137

Veraart, J., Novikov, D. S., Christiaens, D., Ades-aron, B., Sijbers, J., & Fieremans, E. (2016). Denoising of diffusion MRI using random matrix theory. *NeuroImage*, *142*, 394–406. https://doi.org/https://doi.org/10.1016/j.neuroimage.2016.08.016
